# Identification of Kic1p and Cdc42p as Novel Targets to Engineer Yeast Acetic Acid Stress Tolerance

**DOI:** 10.3389/fbioe.2022.837813

**Published:** 2022-03-25

**Authors:** Hong-Qi Chen, Qi Xing, Cheng Cheng, Ming-Ming Zhang, Chen-Guang Liu, Verawat Champreda, Xin-Qing Zhao

**Affiliations:** ^1^ State Key Laboratory of Microbial Metabolism, Joint International Research Laboratory of Metabolic and Developmental Sciences, School of Life Sciences and Biotechnology, Shanghai Jiao Tong University, Shanghai, China; ^2^ National Center for Genetic Engineering and Biotechnology, Pathumthani, Thailand

**Keywords:** *Saccharomyces cerevisiae*, proteome analysis, stress tolerance, zinc sulfate, *KIC1*, *CDC42*

## Abstract

Robust yeast strains that are tolerant to multiple stress environments are desired for an efficient biorefinery. Our previous studies revealed that zinc sulfate serves as an important nutrient for stress tolerance of budding yeast *Saccharomyces cerevisiae*. Acetic acid is a common inhibitor in cellulosic hydrolysate, and the development of acetic acid-tolerant strains is beneficial for lignocellulosic biorefineries. In this study, comparative proteomic studies were performed using *S. cerevisiae* cultured under acetic acid stress with or without zinc sulfate addition, and novel zinc-responsive proteins were identified. Among the differentially expressed proteins, the protein kinase Kic1p and the small rho-like GTPase Cdc42p, which is required for cell integrity and regulation of cell polarity, respectively, were selected for further studies. Overexpression of *KIC1* and *CDC42* endowed *S. cerevisiae* with faster growth and ethanol fermentation under the stresses of acetic acid and mixed inhibitors, as well as in corncob hydrolysate. Notably, the engineered yeast strains showed a 12 h shorter lag phase under the three tested conditions, leading to up to 52.99% higher ethanol productivity than that of the control strain. Further studies showed that the transcription of genes related to stress response was significantly upregulated in the engineered strains under the stress condition. Our results in this study provide novel insights in exploring zinc-responsive proteins for applications of synthetic biology in developing a robust industrial yeast.

## Introduction

Zinc (Zn^2+^) is an important nutrient for various living organisms that plays a vital role in a large variety of cellular metabolic processes. Zinc exerts structural, catalytic, and regulatory roles for cell growth and metabolism of proteins, lipids, and nucleic acids. Zinc is required by multiple enzymes and zinc finger proteins that are important for transcription regulation (Maret, 2017; Cuajungco et al., 2021). In budding yeast *Saccharomyces cerevisiae*, zinc is the cofactor of several alcohol dehydrogenases (ADHs) and also acts as a structural component for various zinc finger proteins, some of which are critical for stress responses (Zhao and Bai, 2012; Eide, 2020). Elucidating how zinc functions in microbial physiology and metabolism are thus of great importance for developing robust strains to achieve desirable fermentation performance.

With the increasing demand for energy as well as concern for climate change and environmental protection, the production of cellulosic ethanol to blend into gasoline has received global interest (Toor et al., 2020; Vieira et al., 2020). *S. cerevisiae* is widely used in cellulosic ethanol production due to its excellent performance in ethanol fermentation (Jansen et al., 2017; Liu et al., 2019). However, economic cellulosic ethanol production remains challenging. One of the most important barriers is the low fermentation efficiency of yeast cells due to the toxicity of inhibitory compounds in cellulosic hydrolysate (Zhao et al., 2016). The inhibitory compounds mainly include weak acids (such as acetic acid and formic acid), furan derivatives including furfural and 5-hydroxymethylfurfural (HMF), and phenolic compounds. These inhibitors are generated from the pretreatment of lignocellulosic biomass (Jönsson et al., 2013; Vanmarcke et al., 2021). Among the inhibitors, acetic acid is very commonly present in various lignocellulosic hydrolysates and is one of the main inhibitors, with the concentration ranging from 1 to 10 g/L (Dong et al., 2013). Acetic acid leads to the acidification of the cytoplasm and affects the activity of intracellular enzymes (Casey et al., 2010), which results in intracellular acidification and insufficient energy supply (Guo and Olsson, 2014; Guaragnella and Bettiga, 2021). In addition, acetic acid also causes endoplasmic reticulum stress and induces the unfolded protein response in *S. cerevisiae* (Kawazoe et al., 2017). Due to the common occurrence of acetic acid toxicity, it is desirable to improve its tolerance of *S. cerevisiae* for efficient lignocellulosic bioethanol production. To combat acetic acid stress, various strategies have been developed, including medium optimization, random mutagenesis, laboratory adaptive evolution, and genetic engineering (Wan et al., 2015; González-Ramos et al., 2016; Zhang et al., 2019a). During the past decade, various functional genes related to acetic acid tolerance were identified. For example, overexpression of *HYP2* (Cheng et al., 2021), *TYE7* (Li et al., 2021), *PRS3* (Cunha et al., 2018), and *WHI2* (Chen et al., 2016); disruption of *RTT109* (Cheng et al., 2016); or downregulating genes encoding subunits of the 19S regulatory particle of the 26S proteasome (Mukherjee et al., 2021) have been employed to improve acetic acid tolerance of *S. cerevisiae*. However, the exact regulatory network underlying acetic acid toxicity remains unclear.

Our previous studies showed that the addition of zinc sulfate improved ethanol fermentation by *S. cerevisiae* in the presence of acetic acid (Wan et al., 2015; reviewed by Cheng et al., 2017). We further explored the possible regulatory mechanisms by metabolic profiling and transcriptomic analyses and identified several key genes for engineering acetic acid tolerance (Wan et al., 2015; Zhang et al., 2017; Zhang et al., 2019b). However, how zinc supplementation influences global protein expression under acetic acid stress is still unclear. We found improved stress tolerance by overexpressing two zinc-containing proteins, namely, the histone H4 methyltransferase Set5p and the zinc finger protein Ppr1p involved in *de novo* pyrimidine biosynthesis (Zhang et al., 2015; Zhang et al., 2017; Zhang et al., 2019b). In addition, we also found that disruption of *ADY2* or overexpression of *ADE17* improved acetic acid tolerance (Zhang et al., 2017; Zhang et al., 2019b). However, so far, no studies have been focused on the impact of zinc sulfate on yeast global protein biosynthesis under stress, and it is of great interest whether proteomic changes by Zn^2+^ supplementation enable the discovery of novel targets for synthetic biology and metabolic engineering of yeast stress tolerance.

Our previous work showed that the addition of 0.03 g/L zinc sulfate improved yeast tolerance to 10 g/L acetic acid (Wan et al., 2015). In this study, we analyzed the comparative proteome in the logarithmic growth stage of *S. cerevisiae* under the same condition. In addition, we demonstrated that Kic1p and Cdc42p, which are involved in cell integrity and polarity, respectively, are involved in the tolerance of acetic acid. The results in the current study thus provide novel insights in zinc biology for the regulation of yeast physiology and metabolism. Our results also suggest alternative strategies for synthetic biology and metabolic engineering of yeast stress tolerance.

## Materials and Methods

### Strains and Culture Medium

All the microbial strains used in this study are listed in Supplementary Table S1. Yeast transformants were screened on yeast peptone dextrose (YPD) agar medium (10 g/L yeast exact, 20 g/L peptone, and 20 g/L glucose) containing 300 μg/ml G418. YPD plates were prepared by adding Bacto agar to a final concentration of 20 g/L. Before ethanol fermentation, yeast cells were cultured in YPD medium and then transferred to fermentation medium (100 g/L glucose, 4 g/L yeast exact, and 3 g/L peptone) with or without acetic acid or mixed inhibitors. The mixed inhibitors mimicking detoxified corn-stover hydrolysate are composed of 4.33 g/L acetic acid, 0.34 g/L formic acid, 0.53 g/L furfural, and 0.36 g/L HMF (Zhang et al., 2015). The composition of the corncob hydrolysate is 0.22 g/L formic acid, 1.45 g/L acetic acid, 10.21 g/L xylose, 100 g/L glucose, and other unknown components. When using corncob hydrolysate, 4 g/L peptone was added into the medium as a nitrogen source.

### Sample Preparation for Proteomic Analysis

For proteomic analysis, the industrial yeast *S. cerevisiae* SPSC01 was inoculated into a 250 ml Erlenmeyer flask containing 100 ml seed medium and cultivated at 30°C, 150 rpm overnight. Next, the strains were deflocculated in 0.1 M sodium citrate buffer (pH 4.5), after which the cells were distributed in several flasks for inoculation. Fermentation was performed in 3 L bioreactors with 1.0 L of the fermentation medium containing 10 g/L acetic acid supplemented with or without 0.03 g/L zinc sulfate, and the initial optical density at 600 nm (OD_600_) was adjusted to around 1.0. The fermentation was carried out at 30°C, 200 rpm, 0.04 vvm, and pH 4.5 and was stopped when the residual sugar of the zinc sulfate addition group was less than 2 g/L. Samples were taken every 12 h, and the corresponding cell growth (dry cell weight), residual sugar, and ethanol were recorded. Yeast cells were collected at the middle log phase in 60 h, then washed three times with cold dH_2_O, and were then used for proteome analysis. Two biological replicates were analyzed, and proteome analysis was performed by The Beijing Genomics Institute (BGI, Beijing, China). The sequencing data were compared with the sequence of *S. cerevisiae* model strain S288C. Enrichment of functional categories among differentially expressed genes was examined using the MIPS Function Catalog (http://mips.gsf.de). Specific gene functions and biological pathways were analyzed based on the information from the *Saccharomyces* Genome Database (SGD) (http://www.yeastgenome.org) and Kyoto Encyclopedia of Genes and Genomes (KEGG) database (https://www.kegg.jp/).

### Construction of Recombinant Yeast Strains

The *PGK1* promoter (PGK1p) was amplified with a pair of primers PPGK1-F/PPGK1-CDC42-R or PPGK1-F/PPGK1-KIC1-R according to the previously described method (Chen et al., 2022). The *KanMX4* cassettes were amplified by PCR with a pair of primers K + CDC42-F/K + P_PGK1_-R or K + KIC1-F/K + P_PGK1_-R, respectively. Then *PGK1p* and the *KanMX4* cassette were used as templates to amplify homologous recombination fragments by overlapping extension PCR (Lu et al., 2018) to replace the native promoter region of *CDC42* or *KIC1* with the pair primers of K + CDC42-F/P_PGK1_-CDC42-R or K + KIC1-F/P_PGK1_-KIC1-R, respectively. Subsequently, the constructed DNA fragments were transformed into the *S. cerevisiae* S288C strain using the chemical method (Bergkessel and Guthrie, 2013). The transformants were screened on YPD plates with 300 μg/ml G418 and were further verified by diagnostic PCR. All primers used are listed in Supplementary Table S2. The selected overexpression transformants of S288C were named S-CDC42 and S-KIC1, respectively.

### Stress Tolerance Assays

Spot assays were performed according to the reference (Zhang et al., 2015) to test the stress tolerance of the mutants and the wild-type strains toward inhibitory conditions including 5 g/L acetic acid (pH value is about 3.7), 5 mM H_2_O_2_, 10% ethanol (v/v), 40°C, 4% lactic acid, and 1.5 g/L furfural, respectively. The inhibitory compounds were added to the medium after the YPD plates were autoclaved. Plates were photographed after 2 days of incubation at 30°C (except for 40°C thermal stress).

### Flask Fermentation

For all the seeds for spot assay, yeast cells were activated twice in the seed culture medium. Single colonies were picked up and cultivated for the first 12 h, and then the culture was transferred into fresh medium and cultivated for another 12 h. After the OD_600_ of all yeast strains were adjusted to be the same, the seed cultures were inoculated into the fermentation medium with the inoculation size of 10% (v/v). The fermentation was performed at 30°C, 150 rpm. Samples were taken every 12 h, and the corresponding cell growth, ethanol, residual sugar, and glycerol were recorded.

### Determination of Metabolites

The concentrations of ethanol, glucose, glycerol, and acetic acid in fermentation samples were determined by high-performance liquid chromatography (HPLC) system with a Bio-Rad Aminex^®^ HPX-87H column, and the elution was carried out at 50°C with 4 mM sulfuric acid and at a flow rate of 0.5 ml/min. The aforementioned compounds were detected and calculated according to previously described methods (Wang et al., 2013).

### Real-Time Quantitative PCR Analysis

The cells of the engineered strains of *S. cerevisiae* and the control strain S288C cultivated in fermentation medium with or without stress were harvested at the log phase. The cell pellets of each sample were immediately stored at −80°C after being washed with sterilized dH_2_O. Total RNA was extracted using a TransZol Plant Kit (TransGen Biotech, Beijing, China) and then reversely transcribed into cDNA using a PrimeScript RT Reagent Kit (TaKaRa, Dalian, China). Subsequently, real-time quantitative PCR (RT-qPCR) was carried out using the SYBR Green qPCR Master Mix (TaKaRa, Dalian, China) with a Real-Time PCR System (Bio-Rad, United States). The *ALG9* gene was used as the reference gene, while relative expression levels were calculated by the 2^−ΔΔCt^ method (Teste et al., 2009). All primers used in this study are listed in Supplementary Table S2.

### Statistical Analysis

All experiments were independently repeated three times, and reproducible results were obtained. The results of the RT-qPCR and fermentation test were expressed as means and standard deviations (SD). Statistical analysis was performed using the Student's *t*-test, and a *p*-value less than 0.05 was regarded as statistically significant.

## Results

### Effects of Zinc Sulfate Addition on Global Protein Expression Under Acetic Acid Stress

**FIGURE 1:**
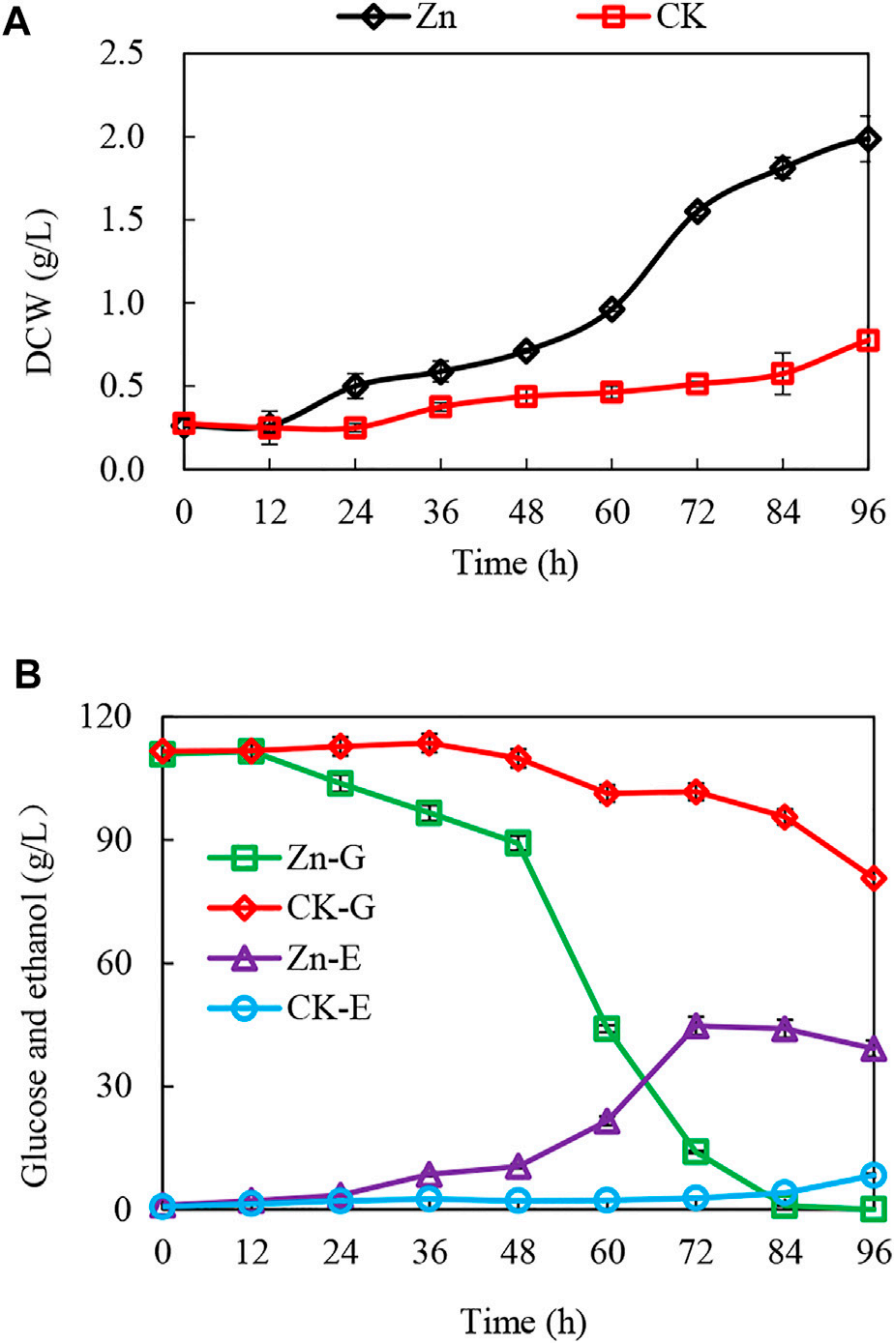
Enhanced growth and ethanol fermentation by zinc sulfate addition under acetic acid stress and interaction of the differentially expressed proteins. **(A,B)** The growth curve, glucose consumption, and ethanol production with or without zinc sulfate supplement under the acetic acid stress. CK-G and CK-E, Zn-G and Zn-E indicate the glucose consumption and ethanol production without or with zinc sulfate supplementation in the presence of 10 g/L acetic acid.

**FIGURE 2:**
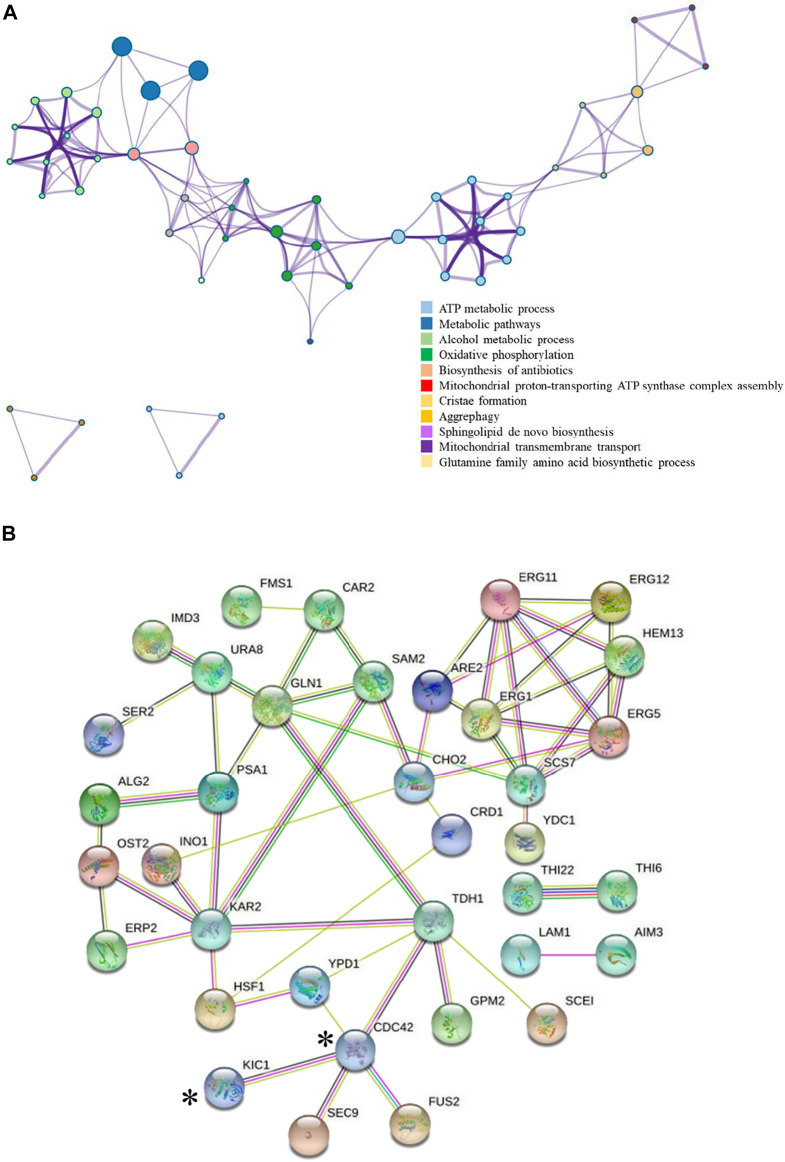
Interaction map of the differentially expressed proteins. **(A)** Interaction of the differentially expressed proteins colored by cluster proteins, where nodes that share the same cluster proteins are typically close to each other. The protein–protein interaction was analyzed by Metascape, and only the data supported by the literature were selected. **(B)** Upregulation proteins interaction network. 
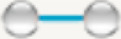
: Known interactions from curated databases. 
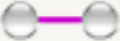
: Known interactions that were experimentally determined; predicted interactions by gene neighborhood (
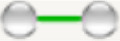
), gene fusions (
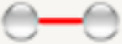
), and gene co-occurrence (
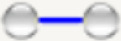
). 
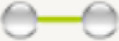
: Text mining. 
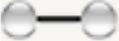
: Co-expression. 
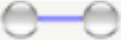
: Protein homology.

The addition of 0.03 g/L zinc sulfate enhanced ethanol fermentation in *S. cerevisiae* SPSC01 (Figure 1). Analysis of the differentially expressed proteins revealed a total of 107 proteins with significant changes (fold change >1.2 or <0.8) (Supplementary Data Sheet S1). The enriched gene ontology (GO) terms of the upregulated and downregulated proteins are shown in Supplementary Figure S1. For the upregulated proteins, the functions in the alcohol biosynthetic process, metabolism of amino acids and derivatives, and ergosterol metabolic process were enriched. Interestingly, most of the downregulated proteins function in cellular respiration, tricarboxylic acid (TCA) cycle pathway, oxidation phosphorylation, and mitochondrial proton-transporting ATP synthase complex assembly. When the protein interaction network was analyzed, it was found that proteins involved in ATP metabolic process, oxidative phosphorylation, and alcohol metabolic process were clustered (Figure 2A).

Acetic acid can cause intracellular acidification and change the intracellular proton gradient, which leads to the inversion of intracellular ATP synthase proton pump and insufficient intracellular energy supply (Guo and Olsson, 2014). We found that nine proteins related to ATP synthase were downregulated by zinc sulfate addition, including Atp20p, Atp4p, Atp19p, Atp7p, Atp15p, Atp16p, Atp5p, Atp3p, and Atp2p, although not very dramatically (Supplementary Table S4). In addition, Cox7p, Cox6p, and Cox12p, which are components of mitochondrial inner membrane electron transport chain, were also downregulated. Considering that energy production is closely related to carbon metabolism, we then further analyzed proteins related to central carbon metabolism.

### Effects of Zinc Sulfate Addition on Proteins Involved in Carbon Metabolism

Differentially expressed proteins involved in the glycolytic pathway, PP pathway (pentose phosphate pathway), and TCA pathway are shown in Supplementary Figure S2. Tdh1p is a glyceraldehyde-3-phosphate dehydrogenase (NAD+) and catalyzes the conversion of glyceraldehyde-3-phosphate to 3-phospho-d-glycerol-phosphate during glycolysis and the reverse reaction during gluconeogenesis (Tkach et al., 2012). Expression of Tdh1p was upregulated (1.32-fold) by zinc sulfate addition, together with Gpm2p (1.23-fold), which is a phosphoglycerate mutase family protein. On the other hand, downregulation of six proteins (Cit1p, Dal7p, Aco1p, Idh2p, Idh1p, and Fum1p) that are involved in the TCA pathway was observed. These downregulated proteins may increase the flux of pyruvate into ethanol production, which is consistent with the increased ethanol production by zinc sulfate supplement.

The transcription factor Zap1p is responsible for regulating gene expression under zinc deficiency conditions (Wilson and Bird, 2016). Based on the analysis using SGD and studies in the literature (Wu et al., 2008), many carbon metabolism enzyme-encoding genes, such as *ADH1*, *ADH4*, *ENO1*, *ENO2*, *TKL2*, and *TDH1*, are proved or hypothesized to be Zap1p target genes. Among these genes, we only found that *TDH1* encoding enzyme was elevated by zinc sulfate addition (1.32-fold relative to the non-addition control) in this study, suggesting that there may be new zinc responsive proteins that were not identified as known Zap1p targets or indirect regulation by zinc sulfate plays an important role.

### Effects of Zinc Sulfate Addition on the Expression of Proteins Related to Stress Response and Tolerance

In our previous studies, transcriptome analysis was performed under the same condition used in this study. However, many differentially transcribed genes in the transcriptome data (Chen et al., 2017) were not found to be changed in our proteome results. This indicated that protein biosynthesis and transcription were differentially regulated by zinc sulfate, which is consistent with the previous reports that transcription changes do not necessarily lead to variation in protein biosynthesis (Lee et al., 2011). Nevertheless, some proteins are changed in both the proteome and the transcriptome results, including the upregulated ones, Ino1p, Pet18p, Pho3p, Thi20p, Sam2p, and Cho2p, and the downregulated ones, Gdh1p, Tmt1p, and Pdc5p. Interestingly, the expression of stress responsive protein Hsp12p was downregulated, but its gene transcription was upregulated by zinc sulfate. Among the proteins, expression of Ino1p and Thi20p was significantly upregulated (6.21-fold and 5.74-fold, respectively) by zinc sulfate under acetic acid stress. Thi20p is a trifunctional enzyme required for thiamine biosynthesis, degradation, and salvage (Onozuka et al., 2008). The C-terminal domains of Thi20p and the whole region of Pet18p of *S. cerevisiae* are homologous to bacterial thiaminase II. We tested the effect of overexpressing *PET18* and *SAM2* in *S. cerevisiae* S288C but did not find any difference of the engineered strains in yeast stress tolerance (data not shown). Ino1p is an inositol-3-phosphate synthase that is responsible for the synthesis of inositol phosphates and inositol-containing phospholipids. It was reported that overexpression of *INO1* considerably improved tolerance of *S. cerevisiae* to lignocellulose-derived inhibitors that include acetic acid, furfural, and phenol (Wang et al., 2015). Our results implied that the increased expression of Ino1p by zinc sulfate may contribute to improved growth in the presence of acetic acid.

Some metabolites protect cells from stress damage, such as glycerol and ergosterol (Wang et al., 2014). In this study, we found that ergosterol biosynthesis enzymes were significantly increased in proteome by zinc sulfate addition, such as Erg20p, Erg1p, Erg11p, Erg3p, Erg5p, and Erg4p (Supplementary Figure S3). Our previous study found that the content of ergosterol was 14.6% higher than that of the control with the zinc sulfate addition, and we also demonstrated that increased ergosterol content may be correlated with improved membrane integrity (Zhang et al., 2017).

To further analyze the effect of zinc ions on global protein expression, the proteins containing zinc ions as cofactors or structural components were analyzed (Supplementary Table S3). Six proteins, namely, Rsf2p, Mal33p, Scs7p, Ams1p, Bet2p, and Axl1p, were identified to show significant changes. Among them, Rsf2p, Mal33p, Scs7p, and Ams1p were upregulated, whereas Bet2p and Axl1p were downregulated by zinc sulfate addition. The involvement of these proteins in stress tolerance was further analyzed by literature search. Rsf2p is a zinc-finger protein that regulates gene expression for acid pH resistance (Mira et al., 2009). On the other hand, the deletion of Mal33p and Scs7p encoding genes led to yeast cells being sensitive to visible light-induced stress resistance (Molin et al., 2020). It will be interesting to study the roles of these zinc-containing proteins in acetic acid stress response and tolerance of *S. cerevisiae*.

We further analyzed the potential interaction of the upregulated proteins (Figure 2B) in the comparative proteomic data. Cdc42p was reported to interact with Kic1p and Tdh1p, and Tdh1p is a key enzyme involved in ethanol production (Tkach et al., 2012). Therefore, we are interested in whether Kic1p and Cdc42p could promote ethanol fermentation efficiency of *S. cerevisiae* under stress conditions due to their specific functions. Upon zinc sulfate addition under acetic acid stress, the expression of Kic1p and Cdc42p were upregulated 2.13-fold and 1.21-fold, respectively, compared with the non-addition control. Kic1p localizes to the cytoplasm and is involved in yeast cell integrity, signal transduction, and budding cell apical bud growth (Sullivan et al., 1998; Vink et al., 2002; Hsu and Weiss, 2013). Cdc42p is involved in the establishment of cell polarity, and it also plays a role late in cell fusion via activation of key cell fusion regulator Fus2p (Johnson and Pringle, 1990; Ydenberg et al., 2012). So far, no related studies have shown that there is an obvious regulatory relationship between zinc ions and the two proteins, namely, Kic1p and Cdc42p. Interestingly, the relationship of Kic1p and Cdc42p with the key mitogen-activated protein kinase (MAPK) Hog1p that regulates multiple stress response was reported previously (Raitt et al., 2000; Janschitz et al., 2019). Hog1p is known to regulate acetic acid stress by affecting the aquaglyceroporin Fps1p (Mollapour and Piper, 2006; Mollapour and Piper, 2007) and the positive regulator Rtg2p, which is the RTG-dependent mitochondrial retrograde signaling that is related to Hog1p function during acid stress (Guaragnella et al., 2019). On the other hand, *KIC1* and *CDC42* were also reported to be the target genes for Yap1p (Salin et al., 2008). Yap1p is responsible for oxidative stress response, and it also functions in ethanol stress tolerance (Gulshan et al., 2011; Zyrina et al., 2017). Although no studies have been reported on the effect of Yap1p in acetic acid tolerance, we have found that acetic acid stress induces reactive oxygen species (ROS) accumulation and that zinc sulfate addition decreased ROS (Wan et al., 2015). Considering the connection of Kic1p and Cdc42p with Hog1p and Yap1p as well as their upregulation by zinc sulfate under acetic acid stress, we were interested whether *KIC1* and *CDC42* may be engineered to improve yeast acetic acid stress tolerance. The involvement of Kic1p and Cdc42p in yeast stress responses was revealed in several previous reports. For example, Kic1p was identified as a novel Hog1p target during hyperosmotic stress response (Janschitz et al., 2019), and Cdc42p is related to sensitivity to heat, cold (Davis et al., 1998), osmotic stress (Basu et al., 2020), and responses to several chemicals (Butcher et al., 2006; Breslow et al., 2008; Sukhai et al., 2013), but how these two proteins influence inhibitor tolerance remains unclear. Therefore, we further studied the effects of these two genes on stress tolerance.

### Effects of Overexpressing *KIC1* and *CDC42* on Yeast Stress Tolerance

**FIGURE 3:**
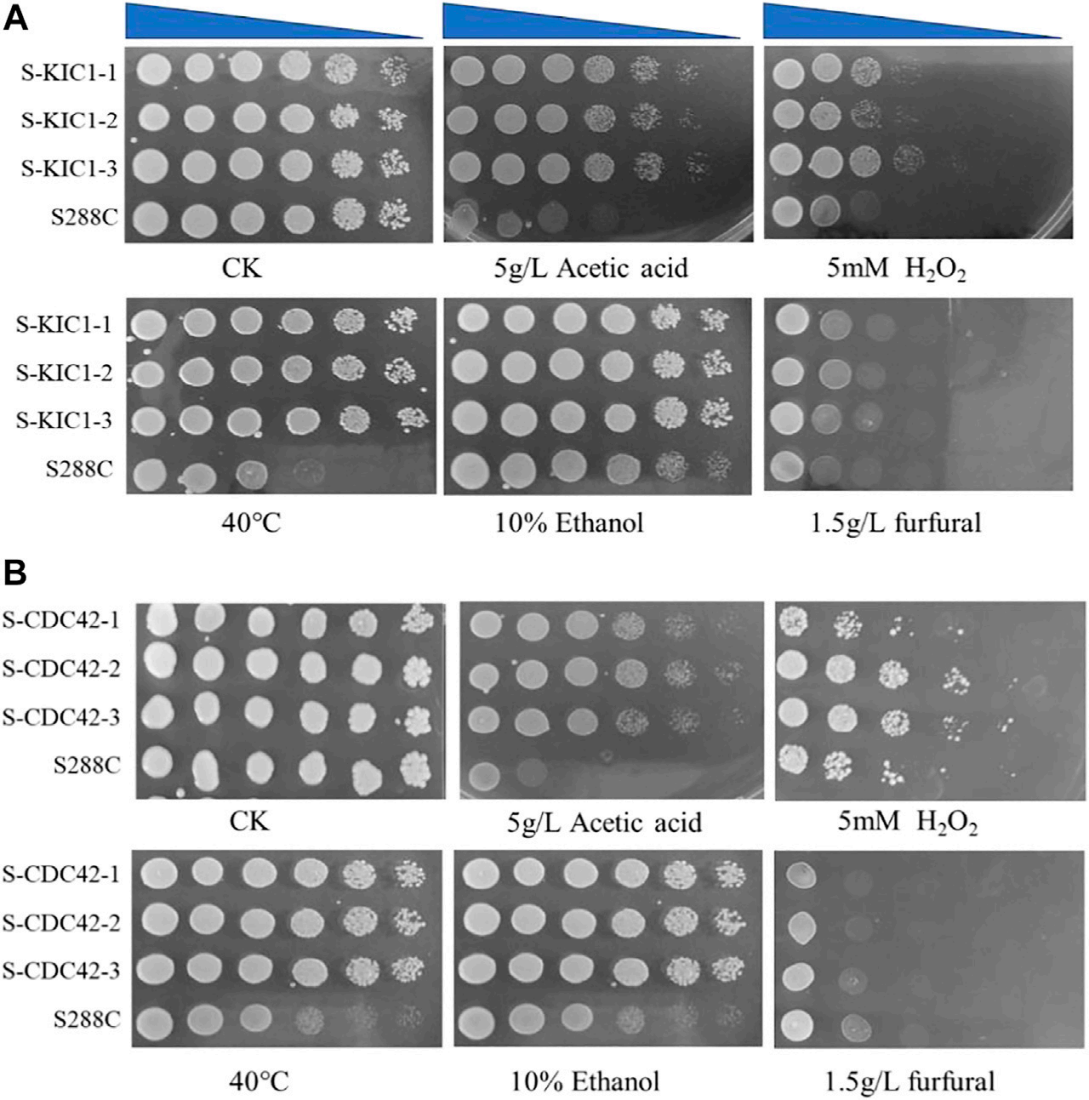
Comparison of stress tolerance of the engineered yeast strains with the parental strain on YPD agar medium. Comparison of tolerance to different stresses of the engineered strain S-KIC1 **(A)** and S-CDC42 **(B)** with that of control strain *S. cerevisiae* S288C. The initial OD_600_ of all strains was about 1.5, and the cells were inoculated on a plate after five-fold serial dilutions. The inhibitor test was performed at 30°C. The inhibitors include 5 g/L acetic acid, 5 mM H_2_O_2_, 10% ethanol, and 1.5 g/L furfural, and the non-addition group was used as a control. For the 40°C thermal tolerance test, 30°C was used as a control condition.

Firstly, a spot assay was performed to test the effect of *KIC1* and *CDC42* on the tolerance of *S. cerevisiae* to various stress conditions. Improved resistance against different stresses in the engineered yeast strains overexpressing the two genes was observed when compared with the parental strain S288C (Figures 3A, B). In addition, the growth of S-KIC1 and S-CDC42 was similar to that of the control strain *S. cerevisiae* S288C when cultured under stress-free conditions. Specifically, S-CDC42 and S-KIC1 showed better growth than that of the control strain when cultured at 40°C or in the presence of 5 g/L acetic acid, and improved growth performance was also observed when the two engineered strains were challenged by 5 mM H_2_O_2_. Together, we revealed that *KIC1* and *CDC42* overexpression exerts significant promoting effects on yeast growth under various stresses in agar plates.

Subsequently, ethanol fermentation in the presence of 5 g/L acetic acid (initial pH at ∼3.5) was further investigated in shake flask culture. The two mutant strains showed similar performance in the presence of acetic acid (5 g/L). S-KIC1 and S-CDC42 showed better ethanol fermentation when compared with S288C under acetic acid stress (Table 1). Specifically, the fermentation time was shortened by 12 h in S-KIC1 and S-CDC42, leading to up to 52.99% higher ethanol productivity than that of the control strain, while the ethanol yield among these strains was comparable (Table 1). In addition, S-KIC1 and S-CDC42 also showed higher glycerol production than the wild-type strain.

**TABLE 1:** Ethanol production performance of the engineered yeast strains under the stresses of 5 g/L acetic acid or in the presence of mixed inhibitors.

Parameter	5 g/L acetic acid			Mixed inhibitors		
	CK	K	C	CK	K	C
*T* (h)	36	24	24	48	36	36
*G* (OD/h/L)	0.74 (±0.08)	0.77 (±0.02)	0.81 (±0.01)	0.58 (±0.01)	0.60 (±0.02)	0.65 (±0.09)
*S*_R_ (g/L)	0.40 (±0.07)	0.86 (±0.15)	1.15 (±0.90)	0.19 (±0.03)	2.35 (±0.04)	0.36 (±0.01)
*E*_p_ (g/L)	42.30 (0.29)	41.84 (±0.28)	43.03 (±0.15)	42.4 (±1.68)	43.08 (±0.49)	43.57 (±0.10)
*G*_p_ (g/L)	0.99 (±0.03)	1.56 (±0.01)	1.60 (±0.07)	1.19 (±0.07)	1.60 (±0.04)	1.56 (±0.05)
*Q* (g/L/h)	1.17 (±0.29)	1.74 (±0.28)	1.79 (±0.15)	0.88 (±0.15)	1.20 (±0.10)	1.21 (±0.12)
*Y*_E/S_ (g/g)	0.42 (±0.09)	0.42 (±0.08)	0.43 (±0.09)	0.42 (±0.08)	0.44 (±0.08)	0.44 (±0.09)

Note: *T*, fermentation time; *G*, growth rate; *S*_R_, residual glucose; *E*_P_, ethanol produced; *G*_P_, glycerol produced; *Q*, ethanol productivity; *Y*_E/S_, ethanol yield, g(ethanol)/g(glucose/sugars); CK, S288C; K, S-KIC1; C, S-CDC42. Three independent experiments were performed, and reproducible results were obtained. The data with SD from three replicates in one of the experiments are shown here.

### The Expression of Stress Response-Related Genes Was Significantly Increased in the Engineered Yeast Strains

To better understand the role of *CDC42* and *KIC1* in the adaptation of yeast cells to lignocellulose-derived inhibitors, we evaluated the performance of S-KIC1and S-CDC42 using the mixed inhibitors (0.34 g/L formic acid, 4.33 g/L acetic acid, 0.53 g/L furfural, and 0.36 g/L HMF) that mimicked the composition of corn stalk hydrolysates. There was a different performance of ethanol fermentation of S-KIC1 and S-CDC42 compared with the control strain S288C under the mixed inhibitors stress. All strains grew poorly due to the strong inhibition of mixed inhibitors, but both engineered strains showed 12 h shorter lag phase, and the strain S-CDC42 showed clearly improved growth than that of S-KIC1 and S288C.

**FIGURE 4:**
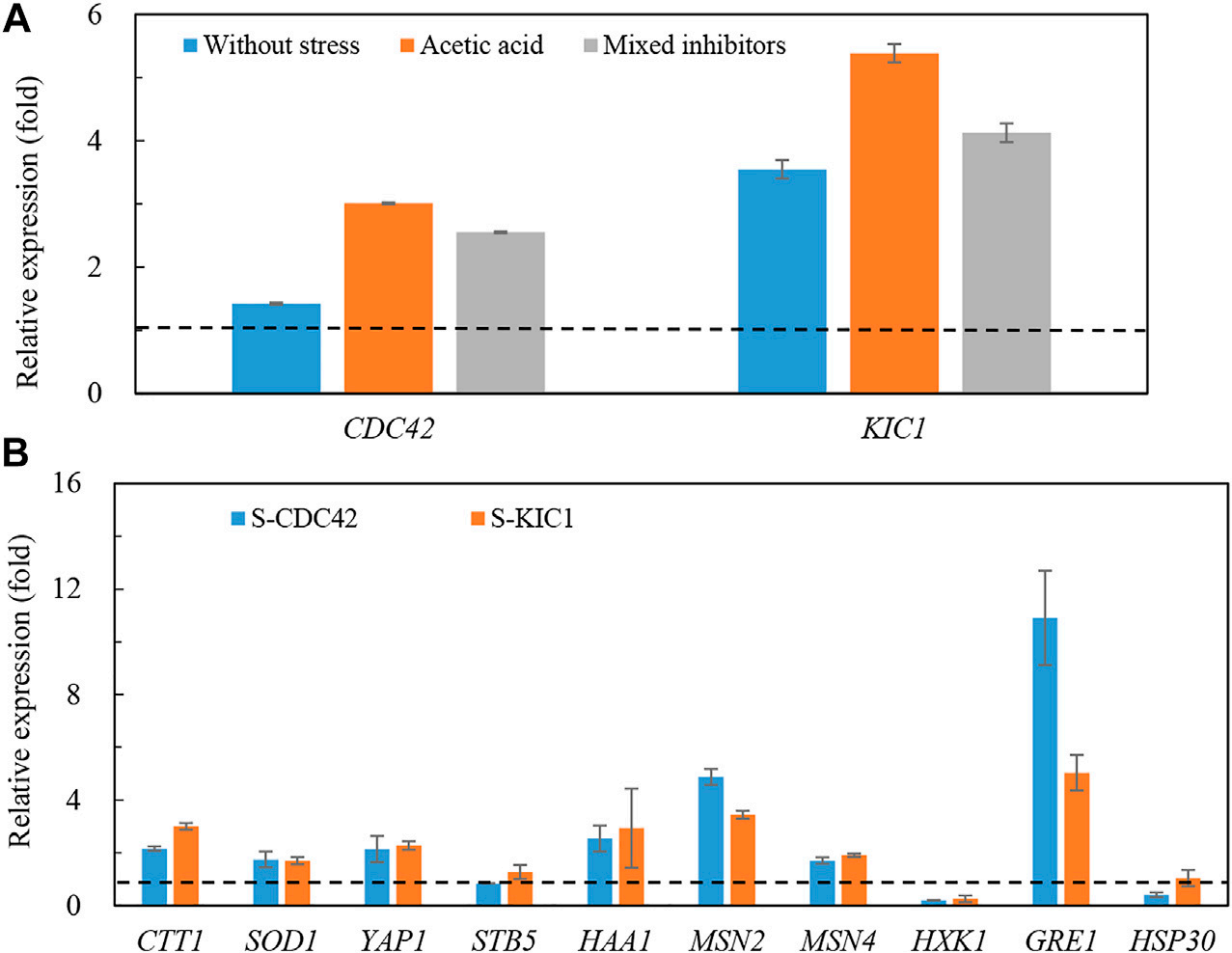
Transcription of genes in the engineered yeast strains and the control strain grown in the mixed inhibitors. The relative transcription of *CDC42* and *KIC1* **(A)** and stress-related genes **(B)** under mixed inhibitors stress was determined. The yeast strains were grown in YPD liquid medium containing the mixed inhibitors, and total RNA was extracted from yeast cells at the log phase. More details are presented in the main text in *Materials and Methods*. The dotted lines indicate one-fold, which is the same level of the control level.

We further explored changes in gene transcription in the yeast strains under stress. As shown in Figure 4, transcription levels of the two genes were more upregulated in the engineered yeast strains than that of the control strain (Figure 4A). Specifically, the transcription of *CDC42* and *KIC1* reached more than three and five times higher than that of the control strain under acetic acid stress, respectively. Higher expression of *CDC42* and *KIC1* was also found under the mixed inhibitors stress. At the same time, we also studied the expression of stress-related genes in the engineered yeast strains (Figure 4B). Transcription levels of *CTT1* (Calabrese et al., 2019), *GRE1* (Garay-Arroyo and Covarrubias, 1999), *HAA1* (Mira et al., 2011), *YAP1* (Okazaki et al., 2007), *MSN2*, and *MSN4* (Hasan et al., 2002) were increased in the two engineered strains under mixed inhibitors stress, and *GRE1* transcription was the most significantly improved. In contrast, no significant changes were observed in *HSP30* (Samakkarn et al., 2021) and *STB5* (Larochelle et al., 2006), respectively. The changes of most of the detected genes except *GRE1* were almost the same in the two engineered strains, and the expression of *GRE1* was higher in S-CDC42 than that in the S-KIC1 strain. These results implied that the mechanisms of enhancing stress tolerance may be different in the two engineered strains.

### Effects of *CDC42* and *KIC1* Overexpression on Ethanol Fermentation With Corncob Hydrolysate

**FIGURE 5:**
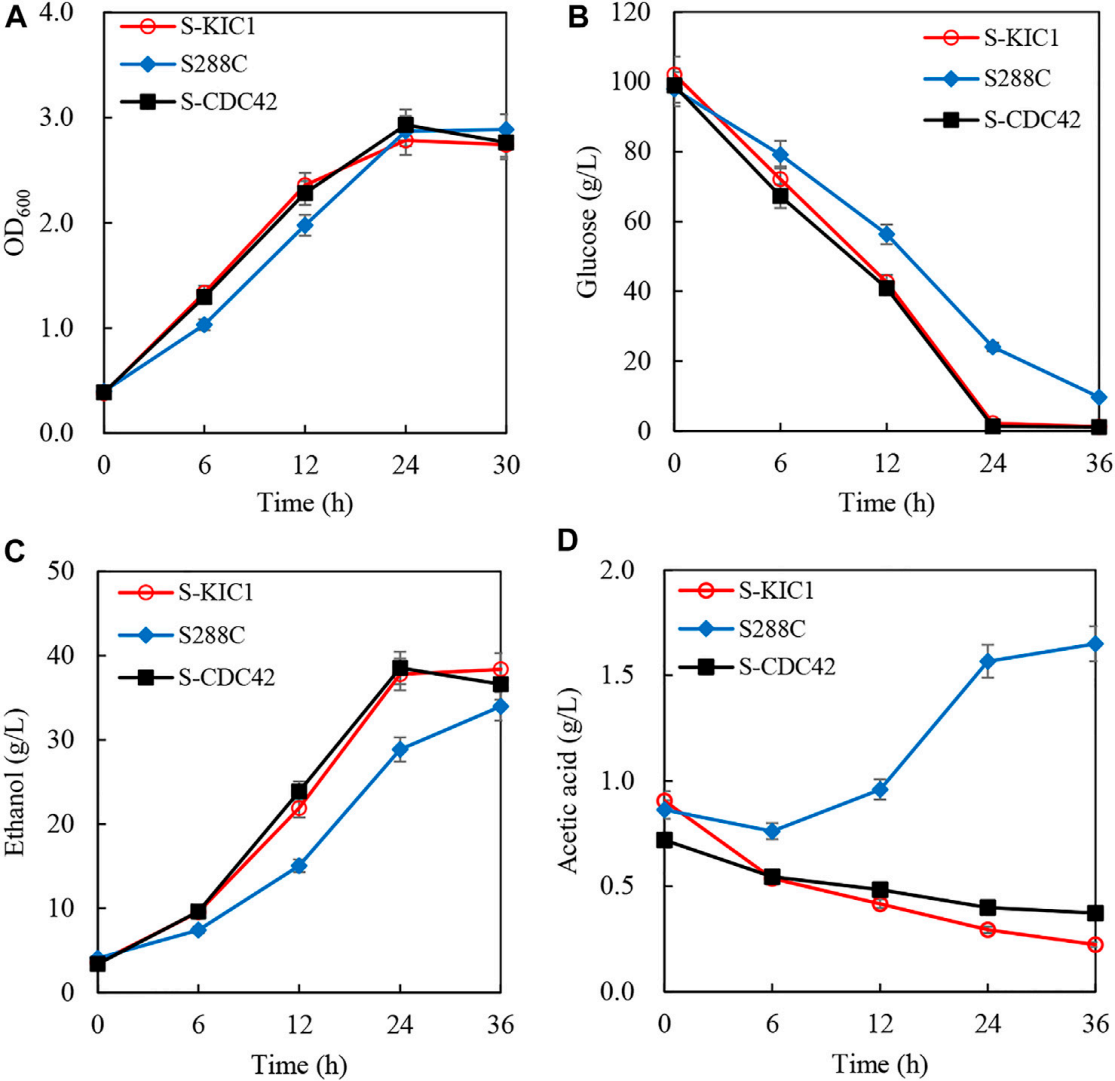
Increased ethanol fermentation from corncob hydrolysate by engineering expression of *KIC1* or *CDC42*. The curve of growth **(A)**, glucose consumption **(B)**, ethanol production **(C)**, and acetic acid content **(D)** were recorded. Yeast strains were activated twice in seed culture medium and then inoculated into the corncob hydrolysate with the inoculation size of 10% (v/v) (initial OD_600_ about 0.1). The fermentation was performed at 30°C with shaking at 150 rpm. Samples were taken every 6 h, and the corresponding cell growth, residual sugar, and ethanol were quantified.

To better evaluate the effects of overexpressing *CDC42* and *KIC1* on the utilization of cellulosic hydrolysate, corncob hydrolysate was used to investigate the ethanol fermentation performance of the engineered yeast strains. There is no difference in the growth of S-CDC42 and S-KIC1 compared with S288C (Figure 5A). However, the engineered strains completely consumed glucose within 24 h, faster than the wild-type strain S288C (Figure 5B). The ethanol production of wild-type strain is 28.8 g/L in 24 h, which is 10 g/L lower than the strains overexpressing *CDC42* and *KIC1* (Figure 5C). The ethanol productivity of S-CDC42 and S-KIC1 reached about 1.60 g/L/h, increased by more than 33% compared with the wild-type S288C. Interestingly, the two engineered strains consumed acetic acid, but the parental strain S288C produced acetic acid as a by-product (Figure 5D).

## Discussion

Acetic acid is the main inhibitor derived from pretreatment of lignocellulosic feedstocks, which could dramatically affect fermentation performance by repressing cell growth and decreasing ethanol production rate and yield (Jönsson et al., 2013; Zhao et al., 2016). The zinc ion is an important cofactor of many proteins in cells (Maret, 2011), and it plays an important role in many biological metabolic processes. The importance of zinc nutrients in ethanol fermentation has been widely recognized due to its role as a cofactor for alcohol dehydrogenase Adh1p (Zhao and Bai, 2012). However, the regulation of proteome by zinc, especially its antioxidant effect, is not well studied, which limited our understanding of the important roles of zinc in cell metabolism. In this study, we found various proteins involved in glycolysis, TCA cycle, transcription regulation, signal transduction, and cellular transport function that are responsive to zinc sulfate addition under acetic acid stress (Figure 2 and Supplementary Figure S1), indicating that zinc acts as a global regulation factor in *S. cerevisiae* by altering carbon flux to improve ethanol production under stress.

Kic1p is a protein kinase that belongs to the PAK/Ste20 family, and it is the component of the RAM (regulation of Ace2p activity and cellular morphogenesis) signaling pathway (Sullivan et al., 1998; Hsu and Weiss, 2013). Until now, nothing is known on the involvement of Kic1p in yeast stress tolerance, and our current work for the first time revealed the effects of Kic1p on tolerance of yeast cells to various inhibitory conditions, including high temperature, acetic acid stress, and oxidative stress. It will be of importance to further elucidate how *KIC1* overexpression remodels the related cell signaling network for improved stress tolerance. *CDC42* is an essential gene that encodes a small GTPase in the Rho/Rac subfamily of Ras-like GTPases that is responsible for the establishment and maintenance of cell polarity (Johnson and Pringle, 1990). However, the relationship between *CDC42* and acetic acid stress remains unclear. It was reported that the functions of both Kic1p and Cdc42p are related to the MAPK Hog1p: Specific residues of Kic1p (Thr^1073^ and Ser^511^) can be directly or indirectly phosphorylated by Hog1p during 0.5 M NaCl shock treatment (Srivas et al., 2016; Janschitz et al., 2019), whereas Cdc42p locates upstream of Hog1p in the MAPK pathway. Hog1p is involved in the regulation of stress response of various stresses, such as hyperosmotic stress, acid pH resistance, acetic acid stress, and oxidation stress (Westfall et al., 2004; Mollapour and Piper, 2006). Therefore, we hypothesize that overexpression of *CDC42* and *KIC1* may affect the cell signaling pathway involving Hog1p. Although both *KIC1* and *CDC42* are involved in bud growth, we did not observe differences of the engineered strains in cell morphology when compared to the wild-type strain (data not shown). The in-depth mechanism of Kic1p and Cdc42p for improving acetic acid tolerance needs to be further studied. In our recent studies, overexpression of *HOG1* was proved to improve yeast growth and ethanol productivity under acetic acid stress (Ye et al., 2022). It will be interesting to further study how these two proteins function together with Hog1p to regulate acetic acid stress tolerance in *S. cerevisiae*.

It should be noted that the transcription levels of *KIC1* and *CDC42* did not change significantly by zinc sulfate addition under acetic acid stress, suggesting that regulations other than the well-studied transcription level are important to yeast stress tolerance, and it is of great importance to integrate transcriptomic and proteomic data for mechanisms studies on stress tolerance. In addition, it will be interesting to elucidate how Kic1p and Cdc42p are regulated at translation or post-translational levels under stress conditions. On the other hand, although we only focused on Kic1p and Cdc42p in this study, our proteomic results also provide the basis for the exploration of other zinc responsive genes for the synthetic biology design of robust yeast strains with improved stress tolerance. It should be noted that not only the proteins can be used to engineer the strain, the zinc-responsive promoters of the proteins may also be explored for rational design, which will be the focus of our future studies.

Taken together, our results provide novel insights for the understanding of zinc ion as a stress protectant for the eukaryotic model *S. cerevisiae*. To our best understanding, this is the first report on the remodeling of protein expression by zinc sulfate, and our results are also the first to reveal the important role of genes related to cell integrity and cell polarity in acetic acid stress tolerance. In addition, our work also promotes the construction of yeast cell factories with better performance in biorefineries using novel genetic elements related to cell morphology and stress signaling.

### Associated Content

The mass spectrometry proteomics data have been deposited to the PRIDE Archive (http://www.ebi.ac.uk/pride/archive/) via the PRIDE partner repository with the data set identifier PXD015459.

## Data Availability

The original contributions presented in the study are included in the article/Supplementary Material, further inquiries can be directed to the corresponding author.

## Supplementary Material

Supplementary Data Sheet S1 Differentially expressed proteins.

Supplementary Figure S1 Pathway enrichment for the differentially expressed genes in the proteome data. Zn/CK represents genes or proteins changed with 10 g/L acetic acid and 0.03 g/L zinc sulfate addition.

Supplementary Figure S2 Differentially expressed proteins related to central carbon metabolic pathway under acetic acid stress by zinc sulfate supplement. The upregulated and downregulated proteins are marked in red and blue, respectively.

Supplementary Figure S3 Protein expression related to ergosterol **(A)** and arginine synthetic pathway **(B)** by zinc sulfate supplement.

Supplementary Table S1 Yeast strains used in this study.

Supplementary Table S2 Primers used in this study.

Supplementary Table S3 Differentially expressed proteins that contain zinc ions in their structures.

Supplementary Table S4 Changed proteins in the proteome involved in ATP synthetic pathway.
