# Supplementary figures and images for: Identification of Kic1p and Cdc42p as Novel Targets to Engineer Yeast Acetic Acid Stress Tolerance
Source: Front Bioeng Biotechnol. 2022 Mar 25;10:837813. doi: 10.3389/fbioe.2022.837813 (PMC8992792; doi:10.3389/fbioe.2022.837813)

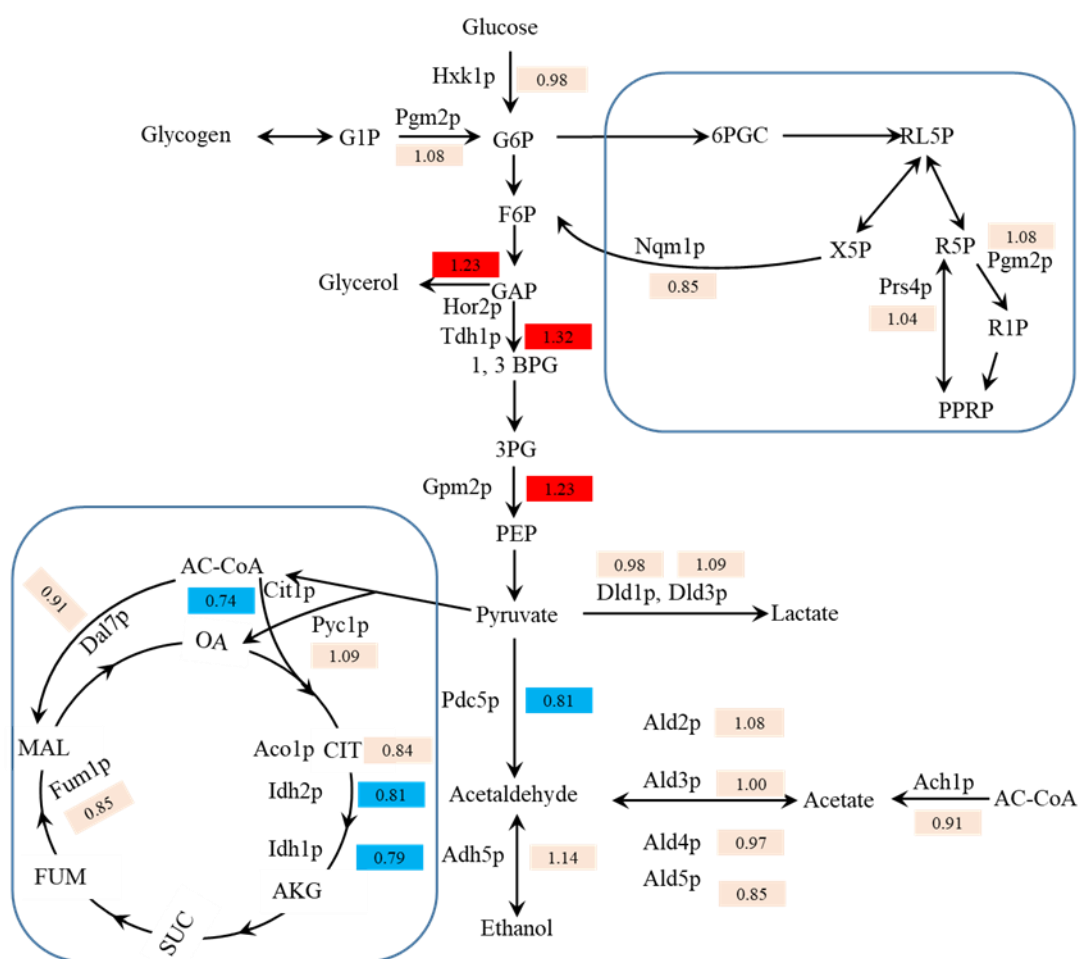

Fig. S2

Supplement: Supplementary file 1 [file Image2.pdf]

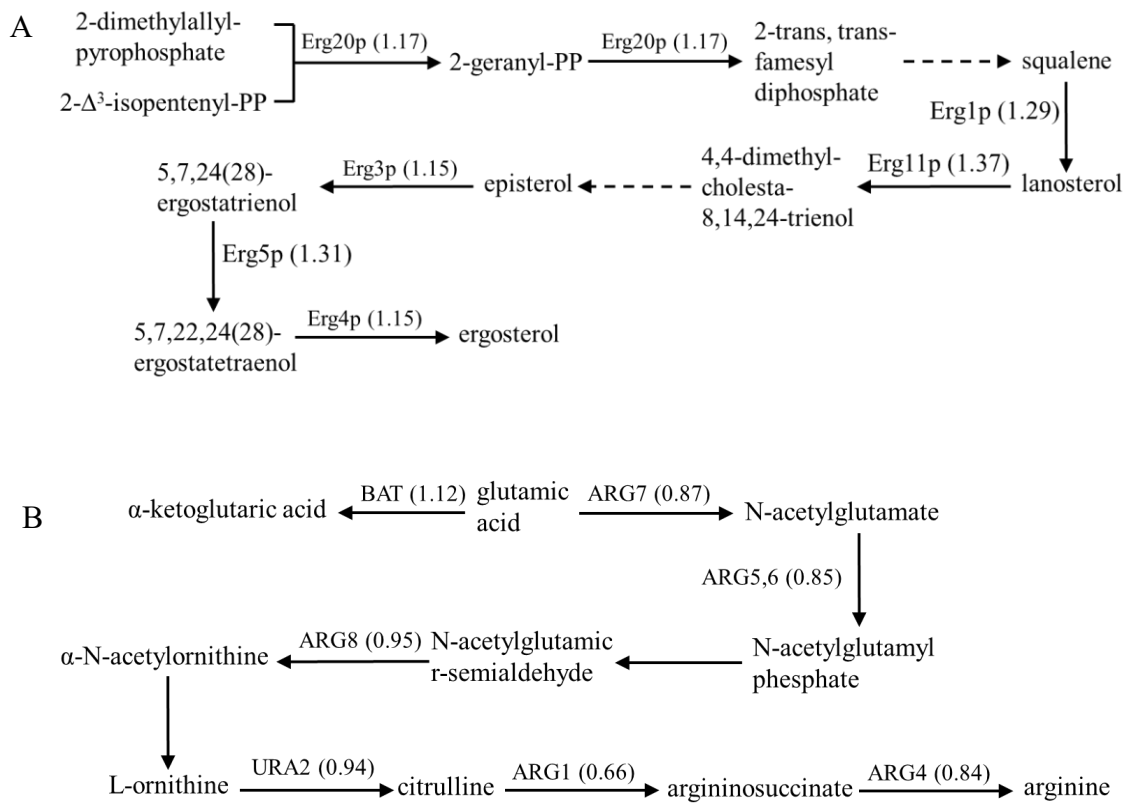

**Fig. S3**

Supplement: Supplementary file 2 [file Image3.pdf]

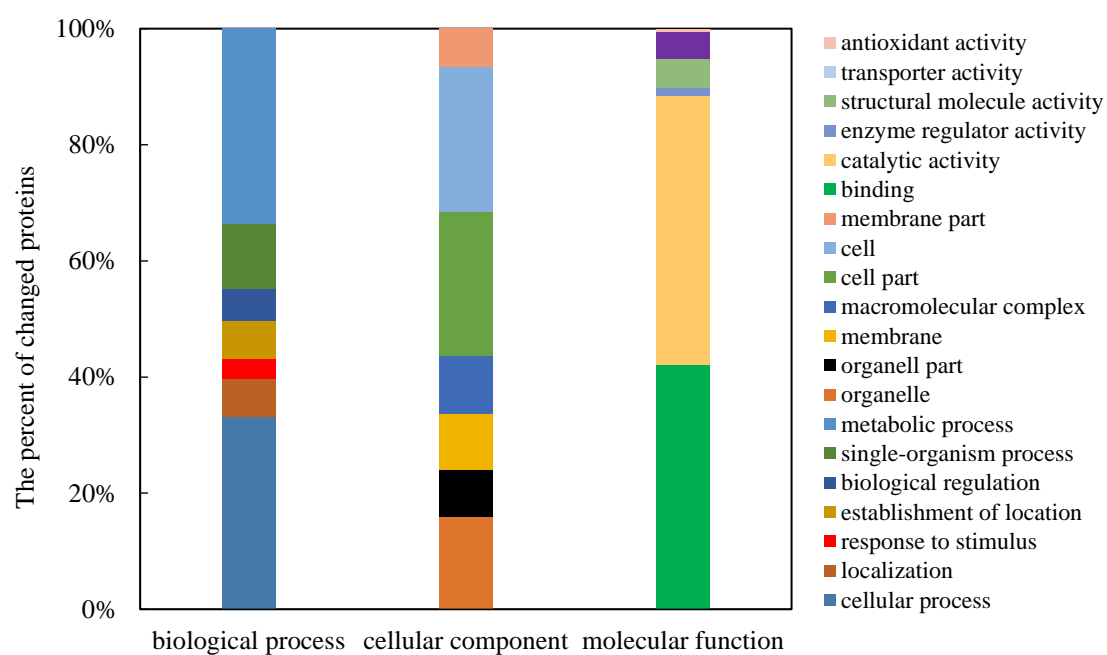

**Fig. S1**

Supplement: Supplementary file 8 [file Image1.pdf]
