# Supplementary material for: Identification of Kic1p and Cdc42p as Novel Targets to Engineer Yeast Acetic Acid Stress Tolerance
Source: Front Bioeng Biotechnol. 2022 Mar 25;10:837813. doi: 10.3389/fbioe.2022.837813 (PMC8992792; doi:10.3389/fbioe.2022.837813)
Supplement: Supplementary file 3 [file Table2.docx]

**Table S2** Primers used in this study

| Primers | 5’ to 3’ primer sequences | | | Purpose |
| --- | --- | --- | --- | --- |
| K+P_PGK1_-R | | TTAGAAAAACTCATCGAGC | Amplification of *KanMX4* and *PGK1* | |
| P_PGK1_-F | | CAGTTTCATTTGATGCTCGATGAGTTTTTCTAA ACTGTAATTGCTTTTAGTTG |  |  |
| K+CDC42-F | | TTTTTAAAAAAAGTTGCATTATTTCTATCAACTGTTGTAGCTACGTTACACCTATGGGTAAGGAAAAGACTCA | Amplification of *KanMX4* and *PGK1* | |
| P_PGK1_+CDC42-R | | TTTGTGGAAGAGCTAATACGTTTATTTCTTGTTTTATAAGTTTTGCTTCTCTTGTTTTATATTTGTTGTAAA |  |  |
| K+KIC1-F | | GTTAAGAGAGGTTTACATTACTACAGAGCTAACCCGTGTTGCATGATATCATATGGGTAAGGAAAAGACTCA | Amplification of *KanMX4* and *PGK1* | |
| P_PGK1_+KIC1-R | | TACTGCCTGTTCTCTATACGTGTTTTGCTTGCCAGTCGATGGCGCCCAAATTTGTTTTATATTTGTTGTAAA |  |  |
| K-F | | ATGGGTAAGGAAAAGACTCA | Validation | |
| K-R | | TTAGAAAAACTCATCGAGCAT | Validation | |
| RT-CDC42-F | | CTGGTCGTCGGTACGCAGATTG | For RT-qPCR | |
| RT-CDC42-R | | GGCGGCCACGATAGCTTCATC | For RT-qPCR | |
| RT-KIC1-F | | ACGTGAGCACCGCAAGTCATTC | For RT-qPCR | |
| RT-KIC1-R | | TGCGAGGTTGTTGTGGCATAGC | For RT-qPCR | |
| RT-ALG9-F | | CACCAGACACTCTCTACGCT | For RT-qPCR | |
| RT-ALG9-R | | GGTTGTTGAGTGAAAACGGC | For RT-qPCR | |

Note: The underlined sequences indicated homologous arms. K is short for *KanMX4*.

**Continued Table S2** Primers used in this study

| Primers | 5’ to 3’ primer sequences | | Target genes |
| --- | --- | --- | --- |
| RT-MSN2-F | | GCTGGGGTTACGAAGGAAAGAAGG | *MSN2* |
| RT-MSN2-R | | TGGGACAAATGTGACAGTGGAACG |  |
| RT-MSN4-F | | TCTCAAGTCATCTCCGGCTCAGG | *MSN4* |
| RT-MSN4-R | | TTTAGCGGCAGCAGAGGCATTATC |  |
| RT-HAA1-F | | GTGTGGGCGAAGTTAGCGTTCC | *HAA1* |
| RT-HAA1-R | | TGCAGCGGTTGCGGTTGTAC |  |
| RT-STB5-F | | CGAAGACATCACTGTGCCACTACC | *STB5* |
| RT-STB5-R | | CGTCTAGCTCCTTGCGTAATCCAC |  |
| RT-YAP1-F | | CGCTTGATAACGACAACGACAACG | *YAP1* |
| RT-YAP1-R | | GGCATAGGAGCCGAATGACTTGG |  |
| RT-SOD1-F | | AGCCTGTGGTGTCATTGGT | *SOD1* |
| RT-SOD1-R | | GATCGACAAGGAACACATGGG |  |
| RT-CTT1-F | | GTTGTTTGCCACGCTTGTAA | *CTT1* |
| RT-CTT1-R | | CAAGGAACTCCCAAGCATTC |  |
| RT-HXK1-F | | TGGGTGAATTGTTGCGTCTA | *HXK1* |
| RT-HXK1-R | | CGATTCTTGCTGGGTAGGAG |  |
| RT-HSP30-F | | TTGGACTGGTGTTCAAGCTG | *HSP30* |
| RT-HSP30-R | | CAGGACAAGAACCAGGCAAT |  |
| RT-GRE1-F | | CAGTTTGGCGGTAACGACTT | *GRE1* |
| RT-GRE1-R | | CTTCATCGTCGTCCAACTGA |  |
