# Supplementary material for: Identification of Kic1p and Cdc42p as Novel Targets to Engineer Yeast Acetic Acid Stress Tolerance
Source: Front Bioeng Biotechnol. 2022 Mar 25;10:837813. doi: 10.3389/fbioe.2022.837813 (PMC8992792; doi:10.3389/fbioe.2022.837813)
Supplement: Supplementary file 4 [file Table3.docx]

**Table S3** Differentially expressed proteins that contain zinc ions in their structures

| Protein name | Folds | Zn^2+^ cofactors^*^ | Function from SGD |
| --- | --- | --- | --- |
| Rsf2p | 1.25 | Zinc finger, C2H2 | Involved in regulation of genes required for glycerol-based growth and respiration |
| Mal33p | 1.32 | Zn(2)-C6 | MAL-activator protein; |
| Scs7p | 1.24 | Binds 2 Zn^2+^ per subunit | Sphingolipid alpha-hydroxylase |
| Ams1p | 1.24 | Binds 1 Zn^2+^ per subunit | Vacuolar alpha mannosidase |
| Bet2p | 0.77 | Binds 1 Zn^2+^ per subunit | Required for vesicular transport between the endoplasmic reticulum and the Golgi |
| Axl1p | 0.25 | Binds 1 Zn^2+^ per subunit | Haploid specific endoprotease of a-factor mating pheromone |

^*^Information predicated by UniProtKB (www.uniport.org)
