# Supplementary material for: Identification of Kic1p and Cdc42p as Novel Targets to Engineer Yeast Acetic Acid Stress Tolerance
Source: Front Bioeng Biotechnol. 2022 Mar 25;10:837813. doi: 10.3389/fbioe.2022.837813 (PMC8992792; doi:10.3389/fbioe.2022.837813)
Supplement: Supplementary file 6 [file Table4.docx]

**Table S4** Changed proteins in the proteome involved in ATP synthetic pathway

| Proteins | Function | Folds | |
| --- | --- | --- | --- |
| Atp20p | Subunit g of the mitochondrial F1F0 ATP synthase | | 0.80 |
| Atp4p | Subunit b of the stator stalk of mitochondrial F1F0 ATP synthase | | 0.79 |
| Atp19p | Subunit k of the mitochondrial F1F0 ATP synthase | | 0.80 |
| Atp7p | Subunit d of the stator stalk of mitochondrial F1F0 ATP synthase | | 0.80 |
| Atp15p | Epsilon subunit of the F1 sector of mitochondrial F1F0 ATP synthase | | 0.76 |
| Atp5p | Subunit 5 of the stator stalk of mitochondrial F1F0 ATP synthase | | 0.77 |
